# Autotransplantation of the Lower Posterior Teeth: A Comprehensive Review

**DOI:** 10.7759/cureus.27875

**Published:** 2022-08-11

**Authors:** Hussain M Algubeal, Abdullah F Alanazi, Abdulaziz S Arafat, Bader Fatani, Ahmad Al-Omar

**Affiliations:** 1 Dentistry, College of Dentistry, King Saud University, Riyadh, SAU; 2 Surgery, King Saud University, Riyadh, SAU

**Keywords:** missing teeth, oral surgery, donor tooth, intentional replantation, autotransplantation

## Abstract

A new era in modern dentistry has emerged where tooth loss is no longer an issue as a result of rapid advancements in implantation and alveolar ridge reconstruction. Despite its wide range of indications, autotransplantation is dependent upon careful patient selection and a suitable technique to ensure successful results both functionally and aesthetically. It is possible to restore physiological occlusion, aesthetics, and masticatory function by varying implant length, diameter, surface, and design, along with autogenous, alogenous, alloplastic, or xenogenous bone substitutes. However, none of the technologies that are used in implant dentistry today can adapt to a child’s growing jaw during adolescence. Thus, the young age of the patient restricts implants and creates a challenge for dentists wishing to replace missing teeth. Therefore, tooth autotransplantation can be a good option for treatment. Our objective in this review is to highlight the biological principles required for the successful autotransplantation of teeth. Limits, indications, and prognoses will be analyzed. Hopefully, with increased awareness and acceptance in the dental profession, autotransplantation will become another viable treatment option for those with compromised teeth who still have significant growth potential.

## Introduction and background

Clinicians around the world experience early tooth loss as one of their biggest challenges. Many factors can contribute to early tooth loss, such as endodontic origin diseases, caries, and fractured (non-restorable) teeth. There are many treatment options to manage early tooth loss, which include extraction without replacement and extraction with replacement using an implant-supported crown, fixed dental prosthesis, or removable dental prosthesis [1]. Tooth autotransplantation is considered a valuable treatment option as an alternative to extraction without replacement, implant-supported crowns, or other treatment options [1,2]. Tooth autotransplantation can be defined as the preplanned repositioning of a certain non-erupted, partially erupted, or fully erupted tooth that is done within the same patient [3-5]. Tooth transplantation through autogenous methods has long been used to replace missing teeth. Many surgical techniques were introduced to establish better stability and a greater longevity period for transplanted teeth. Various factors have been reported to be associated with the success of autotransplantation, including patient age, which is considered a risk factor in complete root formation [6,7]. Autotransplanted teeth have many advantages, such as periodontal ligament (PDL) proprioception, continuous skeletal growth, and better aesthetics [1]. However, root resorption and ankylosis are common complications that are reported frequently [6]. Autotransplantation should be done while considering many factors, such as the techniques to be used and biological value, to ensure a higher success rate. By physiological stimulation of the periodontal ligament (PDL), autotransplantation ensures the preservation of alveolar bone quantity [8,9]. The success and survival rate of autotransplantation depend on multiple factors. Thus, in this article, we will review and illustrate the survival and success rate of tooth autotransplantation.

## Review

Indications

Several factors may lead to the need for tooth autotransplantation. Nevertheless, in most cases, teeth are extracted due to advanced levels of caries destruction [10,11]. In adolescents, the first permanent molars emerge very early and are frequently heavily restored [12]. Imbalanced jaw growth and tooth migration can result in abnormal occlusion in young patients when their first molars are lost [13,14]. In this regard, the treatment of these patients should be concerned with preserving the space left by the missing teeth without causing any alteration to the growing jaw. In adolescents, dental implants do not erupt along with adjacent teeth, which results in infraocclusion with aesthetic and functional problems [15]. A wisdom tooth is most commonly transferred to a hopeless molar because it is late in developing compared to other teeth [16].

Another indication for tooth autotransplantation is maxillary incisors, which are most likely to experience trauma; when such an avulsed tooth is brought to the dental office within 24 hours of trauma and in a suitable solution, it can be replanted and splinted for a period of healing [17]. In some cases, even partially damaged teeth (i.e., cracked, chipped, or with broken crowns) could be restored using endodontics and restorative procedures [18]. Even if a tooth is completely lost (i.e., advanced cariogenic destruction or trauma), it could still be replaced with the patient’s own tooth. The size of the crown and the stage of root development of a donor tooth are considered when selecting a tooth [19]. It is appropriate to use mandibular premolars of mesiodistal dimensions to replace central incisors, although crown reconstruction with composite resin or an artificial crown according to anatomy is demanded later. It may be possible to close the posterior space created by harvesting the premolars with a unilateral protraction of the posterior teeth using traditional or mini-implant anchorage techniques [20,21].

Tooth autotransplantation is also suggested for congenital tooth absence [22]. Agenesis of the teeth is often a result of unidentified causes. In roughly 90% of cases, children with agenesis are missing one or two teeth, and only 3% are missing more than two teeth in a quadrant [23]. An absent mandibular third molar is most common, followed by a missing mandibular second premolar and a missing maxilla lateral incisor [24].

It is also possible to undergo autotransplantation if an atypical eruption of the tooth occurs [25,26]. Usually, teeth that are positioned ectopically are exposed surgically, and then, orthodontic treatment is provided. The treatment of severe or ectopic canines (the presence of ectopic canines occurs in approximately 2% of the population) may prove challenging for traditional orthodontic mechanics. In such an instance, autotransplantation of a canine in a more natural orientation would be more expedient and simpler [27].

Contraindications

To achieve successful autotransplantation, a patient must be carefully selected. An acute infection, poor oral hygiene, or chronic inflammation at the recipient site can delay healing and cause infections, resulting in the failure of the transplanted tooth. As a result, resorption of the alveolar ridge may occur at the recipient site if the receptor bed is insufficiently wide. Therefore, autotransplantation should not be considered for these patients [28,29]. Hence, a successful autotransplantation depends on the characteristics of the recipient site and the donor tooth.

Candidate criteria

The success of autotransplantation is largely determined by patient selection. To ensure a successful outcome of autotransplantation, candidates must be in good health, demonstrate excellent dental hygiene, and be agreeable to regular dental care. To ensure predictable results, patients must be able to follow postoperative instructions and be available for follow-up visits; cooperation and understanding are essential factors of success. Most importantly, the recipient site and donor tooth must be suitable [28-33]. Guidelines for chosen transplant cases are demonstrated in Table 1.

**Table 1:** Guidelines chosen by the authors for transplant cases Adapted from [3]

Aspects to consider		Description
Patient aspects	Patient motivation	Motivated patients for surgical procedures followed by root canal therapy
	Consent	A transplant is chosen as the replacement for a missing tooth after all options are discussed
	Medical history	No medical history or immune impairment precludes oral surgery for this patient
Clinical aspects	Oral hygiene	The importance of good oral hygiene and healthy gingiva
	Root configuration	The roots of both extracted and transplanted teeth have similar lengths and shapes, allowing for a good fit in the transplantation site
	Inferior alveolar nerve	Keep away from second molar socket
	Surgical procedure	Keep transplant teeth out of the mouth for as short as possible; store them in saline or milk when removed from the mouth
	Splinting and follow-up	Root canal treatment to begin after the use of flowable composite and wire splint for up to four weeks

Recipient site criteria

To have a successful tooth autotransplantation, the recipient site must be clear of any source of infection, whether acute or chronic, with an adequate amount of keratinized gingiva to help in the stabilization of the transplanted tooth, good vascularity, and adequate bony support in all dimensions [28]. Adequate bony support may not be present in some cases due to various reasons such as tooth aplasia or its early loss. Alveolar ridge resorption and root protrusion through dehiscence may occur in cases of insufficient bony support, so the use of free bone autografts in these cases is highly recommended [4].

Donor tooth criteria

**Figure 1:**
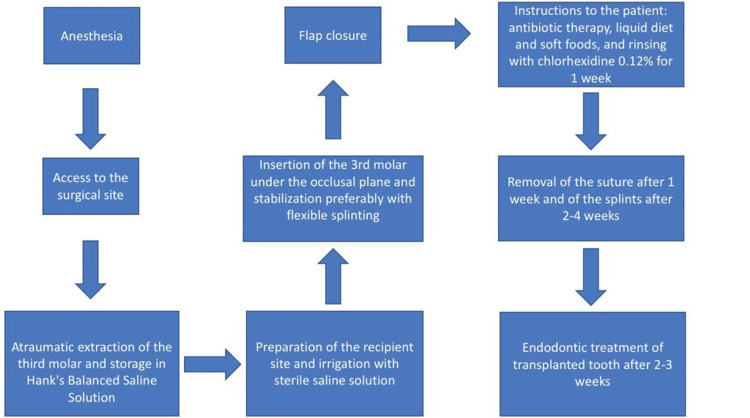
Surgical steps for autotransplantation Adapted from [4]

The donor tooth should be atraumatically extracted to preserve and minimize the damage to the periodontal ligaments around the tooth [1,4], as one of the most important factors that affect the success of a transplanted tooth is the viability of periodontal ligaments [4,5,34]; these contain cells that have the potential to differentiate into cementoblasts, osteoblasts, and fibroblasts, and they have an important role in tissue regeneration [4]. Several studies have indicated that donor teeth with atrophic periodontal ligaments can be easily damaged and have a higher risk of complications [15,28,34]. Reducing the extraoral time of the donor tooth is an important factor to reduce periodontal ligament damage [10]. Increasing the time interval between the extraction and transplantation of the tooth will reduce the prognosis of the procedure [4]. The use of a 3D replica is recommended to reduce this time interval and decrease the number of attempts to position the donor tooth in the recipient site, which will reduce periodontal ligament damage [4,10]. The 3D replica can be used as a guide for preparing the recipient site until it is perfectly fitted; then, extraction of the donor tooth can be done. This can limit the extraoral time of the donor tooth to less than one minute [10]. If extraoral manipulation of the donor tooth is needed, such as root-end resection or root-end filing, maintaining the donor tooth under appropriate storage conditions is important, such as in normal saline or Hank’s Balanced Salt Solution [1]. Regarding the root formation of the donor tooth, transplanted teeth that have incomplete root formation have a 96% rate of pulp healing, while this rate is 15% for teeth that have completed root formation [4,19]. Teeth with a 75% degree of root formation and an immature apex have a higher capacity for revascularization [4]. Many studies show that a tooth with incomplete root formation has a higher success rate after transplantation [4,19]; in contrast to these studies, a 10-year retrospective study was done evaluating 82 cases from 2006 to 2016 and found a higher failure rate among immature teeth compared to matured teeth, and a fully erupted donor tooth was significantly associated with longer tooth survival [34]. Teeth having developed full-length roots and the potential for pulp regeneration (i.e., opening of the apex > 1 mm) have the best-anticipated results [28]. Teeth with abnormal morphology requiring sectioning for extraction should not be considered donors [28]. Figure 1 illustrates the surgical steps for autotransplantation.

Tooth positioning and stabilization

A donor tooth must be positioned at the recipient site with a biological width similar to a tooth that is naturally erupted [35]. To avoid occlusal forces acting on the donor tooth interfering with the healing of the periodontium after transplantation, it is important that the donor tooth be kept out of occlusal contact at the recipient site [8]. Stabilization of the tooth after placement is required; however, the effectiveness of this stabilization on periodontal healing is still controversial [31]. Sutures, ligatures, orthodontic brackets, and composite resins are some of the techniques that may be used to stabilize transplanted teeth [36,37]. The period of immobilization varies from one to six weeks [38-41]. Splinting with rigid splints and up to three months of fixation was thought to cause periodontal regeneration [41,42]. For the procedure to be successful, it is imperative that the splint be chosen carefully [43]. In situations where the donor tooth exhibits reduced stability, a rigid splint is required [44,45]. Inflammatory root resorption (IRR) or ankylosis can occur because of splints, adversely affecting oral hygiene and periodontal regeneration, which can undermine long-term success [46,47]. Occlusion must be examined to ensure that there is no occlusal interference, followed by a determination of what kind of restorations are necessary to improve the occlusion and aesthetic appearance of the tooth crown. To assess the position of the donor tooth after the surgery, as well as before and after splinting, an X-ray is taken before and after the surgery. A surgical dressing is applied to protect the graft against infection during the first 2-3 days of wound healing. Following surgery, this dressing is removed after approximately 3-4 days [39]. In the first week post-operation, it is necessary to provide instructions regarding oral hygiene and diet. Following suture removal, a follow-up appointment is usually scheduled for after 7-10 days [48].

Factors affecting the success of autotransplantation

**Figure 2:**
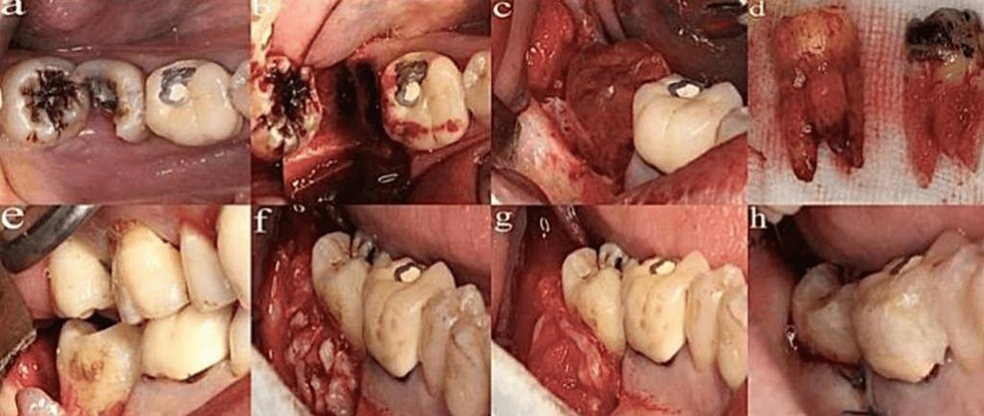
Demonstration of autotransplantation surgical procedure of a third molar in a fresh socket of a second molar a: Hopeless second molar. b: Extraction of a second molar with a fresh socket. c: 3D replica demonstration. d: 3D replica approximately equal to the donor tooth. e: Try-in of the third molar at the fresh socket. f: Autogenous bone grafting buccally and distally. g: Concentrated growth factor membrane applied. h: Suturing of the flap. Reproduced after written permission from [10] (this article is distributed under the terms of the Creative Commons Attribution 4.0 International License (http://creativecommons.org/licenses/by/4.0/), which permits unrestricted use, distribution, and reproduction in any medium)

Several factors can affect the success or survival rate of the tooth after autotransplantation, including the recipient site condition (alveolar bone volume and local inflammation), surgery technique (intraoperative medications and technique used for stabilization), surgical trauma, and stage of root formation. Failure or poor success rates of transplanted molars may be due to the requirement of higher surgical skill, trauma during extraction, and complex root structure or anatomy [10,49]. Healthy periodontal tissue is considered one of the most important factors that affect the success or survival rate of the transplanted tooth. However, the donor tooth remaining outside the oral cavity may damage its periodontium [10]. The most common obtainable and available teeth for autotransplantation are premolars due to orthodontic extractions [49]. One study assessed 19 clinical variables for transplanted teeth, which were divided into three categories. The first category was subject factors, including age, sex, donor tooth maturity, donor tooth position, donor erupted status, donor and recipient relationship, transplantation timing, adjacent marginal bone defect, and recipient position. The second category was procedural factors, including extraction type, extraoral time, bone graft, initial stability, using mineral trioxide aggregate (MTA) in root-end resection or retrofilling, and orthodontic treatment. The third category was postoperative complication factors, including inflammatory root resorption (IRR), ankylosis, and marginal bone loss [34]. Another study assessed the factors influencing the success of autotransplantation in posterior teeth; the results showed that premolar transplantations were more successful than molar transplantations, but success rates were significantly different depending on the surgeon’s skill level [50]. The autotransplantation surgical procedure is shown in Figure 2.

Recommendations suggested for a successful autotransplantation

For a successful autotransplantation, a number of recommendations have been suggested in previous studies [5,30], which include the following: patients should be healthy and medically fit, any infection at the host site should be controlled, postoperative supragingival plaque should be controlled, the donor site should have a normal morphology that should match the recipient site without any complications, half to three-quarters of root formation with an immature root apex (1-mm width of the open apex), atraumatic extraction (preserving bone and periodontal support), less than one minute of extraoral time for the donor tooth, donor tooth should be placed into a fresh extraction site (preferably arranged using a 3D surgical tooth template), adequate fixation (rigid splinting or increased time of fixation of the transplanted tooth can affect its healing outcome), periodontal healing normally accomplished after 7-8 weeks, the diagnosis can be made radiographically as the presence of lamina dura and a continuous space around the root with no root resorption, most transplantation trauma should be avoided, and excellent oral hygiene should be maintained.

Evaluation of success

The main goals of tooth transplantation are the absence of ankylosis and the survival of PDL [51]. The criteria for successfully transplanted teeth are divided into two main groups: radiographic criteria, including a normal width of periodontal space around the reimplanted tooth, a radio-opaque line at the septal bone (lamina dura), no root resorption [19,21,28,52], normal periapical healing [5], no apical infection, and a crown-to-root ratio of less than one to maintain tooth function [21,23], and clinical criteria, including normal movement of the reimplanted tooth, no periodontal pocket, no signs of inflammation, normal function of the tooth, the patient should not feel pain [28,52], positive vitality response (if the tooth was vital), normal root development [53], and normal sound on percussion [52].

Potential complications of autotransplantation

Surface, inflammatory, and replacement resorptions are the three types of root resorption [54]. Table 2 shows a brief description of these three different types of resorptions. Infraocclusion, loss of lamina dura on radiographs, and a “high metallic” percussive sound are all signs of replacement resorption or ankylosis within six months of transplantation. In 1990, Andreasen et al. found an incidence rate of 4.8%, but the study included samples that were stored extraorally for different periods of time [39]. A 10-year follow-up of 162 transplanted premolars reported 7% ankylosis, while 49 third molars showed up to 40% ankylosis [55]. When bacterial contamination occurs and the apex diameter is less than 1 mm, there is a high risk of inflammatory resorption [39]. The necrotic debris stimulates the odontoclasts, which results in progressive resorption of the dentine. It is possible for this condition to occur following an autotransplantation within one month of the procedure; it must be treated through endodontic procedures to remove the inflammatory stimulus (i.e., the infected pulp tissue), which causes the infection [54,56]. Taking care in handling the PDL, having an extraoral time under one minute, and having an immature root apex (1 mm width open apex) are essential to avoid posttransplant complications. Success depends on the experience of the operator and the accuracy of the surgical protocol [57].

**Table 2:** Complications of autotransplantation Adapted from [58]

Types of root resorption	
Surface resorption	As a result of traumatic or other insults to the cementum (e.g., orthodontic movements), small areas of necrosis are developed. Osteoclasts remove this necrotic tissue. In this case, the periodontal ligament has been reestablished after the area of injury has become small enough for the adjacent cementum to grow into the area, and a normal periodontal ligament has developed. There is no loss of root due to this self-limiting process.
Replacement resorption/ankylosis	This results from direct contact between the roots and the bone. In this condition, osteoclasts from the bone resorb the root directly, and new bone is laid down by osteoblasts to replace it. It is the result of an excessively necrotic periodontal ligament. When it starts, it cannot be stopped, and the tooth will eventually fall out.
Inflammatory resorption	Through dentinal tubules, a necrotic and infected pulp communicates with the adjacent periodontal ligament space. Within months, the root resorbs rapidly. To stop this resorption, root canal therapy must be initiated to remove the inflammatory stimulus. Whether cemental or bony (replacement resorption) healing occurs depends upon the size of the necrotic cementum area.

Prognosis of treatment

As far as the success of autotransplantation is concerned, there is a limited amount of evidence that is available. This study was one of the largest studies by Andreasen et al., which included 370 transplanted premolars. The premolars were followed for an average of five years after transplantation and were assessed for their durability. As reported by Andreasen et al., 86% of the cases were normal healing, 13.9% of the cases were successful clinically but with proof of root resorption, and 0.1% were extracted from the patients [39,59,60]. In one of the longest follow-up studies examined by Czochrowska et al., 28 patients with a total of 33 transplanted teeth were followed up for a mean of 26 years after their transplantation. After a period of nine, 10, and 29 years, they reported losing three teeth, and a clinical success rate of 90% was reported. The overall success rate was 79% as four teeth had proof of ankylosis or did not meet the criteria for success due to their conditions [23].

## Conclusions

Tooth autotransplantation is a valuable alternative treatment option that requires a multidisciplinary team approach to restore function and aesthetics to the patient. Many factors can contribute to the failure of the procedure; thus, careful selection of patients, good vascularity, adequate bone support, and viability of the periodontium are important factors to increase the success rate. The stage of root development is also linked to the success rate, with a poor prognosis for a tooth with completed root formation. Endodontic treatment is required for such cases after 2-3 weeks. Root resorption and tooth loss are possible treatment outcomes. Patients are required to commit a careful follow-up after the treatment.
